# Engage Coaching for Lonely Older Adults: Changes in Daily Loneliness and Social Activities

**DOI:** 10.1093/geroni/igaf122.194

**Published:** 2025-12-31

**Authors:** Kimberly Van Orden

**Affiliations:** University of Rochester Medical Center, Rochester, New York, United States

## Abstract

Social disconnection—low engagement, isolation, and loneliness—is associated with poor health, low quality of life, and premature mortality for older adults. However, it is not yet clear which interventions work, especially for older adults who are not homebound (with access to social activities). Identifying mechanisms that account for improvements could help identify effective strategies. This presentation describes a clinical trial testing a brief social engagement coaching program for lonely older adults. Momentary loneliness (perceived social isolation, PROMIS Social Isolation Short Form) and frequency of activities outside the home are assessed via 10 days of ecological momentary assessment (EMA) at baseline, 3-months and 6-months. Currently, 29 out of 30 planned subjects completed baseline EMA protocols; 13 have completed follow-up EMA. The sample has an average age of 72.26 years; 5 subjects are male, 19 female, and 1 intersex. The average number of activities outside the home at each survey instance was less than 1 at baseline (0.55, std 1.10) and increased to 1.25 at 3-months (std 1.74, p = 0.000). Loneliness severity at baseline was 9.04 (std 1.84) and decreased to 8.88 (std 1.85) at 3-months (p = 0.000). Presentation results will include the full sample and 6-month follow-up; effect sizes to illustrate magnitude of effects; and examination of sequential effects in mediation. Results indicate reliable reductions in loneliness and increases in activities outside the home after social engagement coaching. Results suggest that greater activities outside the home may be one mechanism accounting for reduced loneliness in older adults participating in social engagement coaching.

